# High Tumor Mutation Burden (TMB) and a Novel Somatic Mutation in the TREX1 Gene in a Patient with Aggressive and Refractory High-Grade B-Cell Lymphoma: A Case Report

**DOI:** 10.3390/ijms26072926

**Published:** 2025-03-24

**Authors:** Mariia Gusakova, Fedor Sharko, Eugenia Boulygina, Natalia Slobodova, Maria Gladysheva-Azgari, Darima Badmazhapova, Artem Bullikh, Marina Khestanova, Nelli Gabeeva, Tatiana Obukhova, Eugene Zvonkov, Svetlana Tsygankova

**Affiliations:** 1 National Research Center “Kurchatov Institute”, 123182 Moscow, Russia; 2 National Medical Research Center for Hematology, 125167 Moscow, Russia; 3 V.I. Kulakov National Medical Research Center for Obstetrics, Gynecology, and Perinatology, Ministry of Health of the Russian Federation, Akademika Oparina Street, 4, 117997 Moscow, Russia; 4 Faculty of Biology and Biotechnology, HSE University, 101000 Moscow, Russia; 5 Pathology Department, JSC “Medsi”, 143442 Moscow, Russia

**Keywords:** HGBL, NOS, GCB, BCL2/IGH rearrangement, whole-exome sequencing, TMB-High, TREX1

## Abstract

High-grade B-cell lymphoma (HGBL), not otherwise specified (NOS), is a rare entity within the spectrum of B-cell lymphomas. HGBL, NOS remains a diagnosis of exclusion with limited data available on the optimal clinical approach. We report a case of a 67-year-old man with HGBL, NOS with a germinal center B-cell (GCB) immunophenotype. The disease was characterized by an aggressive clinical course, refractory to multiple lines of cytotoxic chemotherapy, immunotargeted treatment, therapy with a PD-1 inhibitor, and haploidentical hematopoietic stem cell transplantation (haplo-*HSCT*). Ultimately, the disease progression led to the patient’s death nine months post-diagnosis. A FISH assay identified a sole genetic rearrangement: BCL2/IGH. Whole-exome sequencing revealed a number of significant somatic mutations, such as TP53 p.C238G, B2M p.L12R, STAT6 p.D419G, STAT3 p.S614R, TREX1 p.T49fs, and CREBBP p.C367Ter, as well as a high focal amplification of the MUC3A gene and the deletion of the short arm of chromosome 17 (del(17p)). An inactivating somatic mutation in the TREX1 gene (p.T49fs) has not been previously described in patients with non-Hodgkin lymphomas. Additionally, our analysis uncovered a key cancer hallmark: tumor genomic instability, manifested as a high tumor mutational burden, which likely contributed to the aggressive disease course.

## 1. Introduction

B-cell lymphomas are malignant hematological diseases that comprise a histologically and molecularly heterogeneous group of various subtypes. The majority of cases are diffuse large B-cell lymphomas (DLBCLs); however, within this classification, high-grade B-cell lymphomas (HGBLs) are distinguished based on specific genetic alterations (MYC, BCL2, and BCL6 translocations) and histological features [1].

In 2016, the WHO introduced a new histomolecular classification, defining HGBL with MYC and BCL2 and/or BCL6 rearrangements (referred to as “double-hit” or “triple-hit” lymphomas, HGBL-DHL/THL) and HGBL, not otherwise specified (NOS). HGBL, NOS encompasses the morphological features of Burkitt lymphoma (BL) and DLBCL and has a blastoid morphology but lacks “double-hit” or “triple-hit” cytogenetics as identified by FISH or conventional karyotyping, preventing its classification into other well-defined subtypes [2,3].

In the fifth edition of the classification (2022) and the latest update of the International Consensus Classification of Lymphoma, the definition of HGBL, NOS was fully retained [4,5]. Meanwhile, HGBL with two or three rearrangements, due to its high molecular and histological heterogeneity, has been redefined as “diffuse large B-cell lymphoma/high-grade B-cell lymphoma with MYC and BCL2 rearrangements”.

Given its aggressiveness and prognosis, HGBL, NOS is considered an intermediate entity between DLBCL and HGBL-DHL/THL. Only a few studies have compared treatment outcomes for HGBL, NOS using different chemoimmunotherapy regimens [6,7]. Currently, there are no standardized clinical treatment algorithms for this malignancy, which is characterized by an aggressive course and resistance to existing therapeutic approaches [8].

In patients with HGBL classified as intermediate- or high-risk according to the National Comprehensive Cancer Network International Prognostic Index (NCCN-IPI), the disease is often highly disseminated, with bone marrow involvement and multiple extranodal lesions [9].

For more than two decades, B-cell lymphomas have also been classified based on their cell of origin. Initially, transcriptome analysis defined two subtypes: activated B-cell (ABC) and germinal center B-cell (GCB) tumors. These subgroups exhibit distinct clinical behaviors and molecular features that reflect differences in disease pathogenesis [10,11]. Currently, the adapted Hans immunohistochemical algorithm is the most widely used method in clinical practice for determining these subtypes [12,13].

Due to the recent classification of HGBL, NOS as a distinct entity separate from the general DLBCL category, as well as its low incidence, its molecular genetic features remain poorly understood. Researchers are currently working to establish connections between the cytogenetic classification of DLBCL and HGBL, the dichotomous classification of B-cell lymphomas into ABC and GCB subtypes, and the molecular genetic profiles of these tumors [5].

Several molecular genetic classifiers have been developed for DLBCL and HGBL. The most comprehensive to date is the LymphGen algorithm, which identifies six well-defined subgroups: MCD, BN2, EZB-MYC, ST2, A53, and N1 [14,15]. Some studies have attempted to correlate the DLBCL/HGBL classification with the ABC/GCB subtypes based on molecular genetic features. A general pattern has emerged: the GCB subtype, particularly HGBL, is associated with the EZB-MYC and ST2 subgroups, while the ABC subtype corresponds to MCD, N1, and BN2. However, due to the high heterogeneity of these tumors, some cases exhibit mixed characteristics and do not fit into a specific molecular subgroup [5].

This paper presents a clinical case of HGBL, NOS, including a detailed description of the treatment regimen and its effects, along with a retrospective analysis of the tumor’s molecular genetic profile based on whole-exome sequencing.

## 2. Case Report

### 2.1. History of the Disease and Treatment

A 67-year-old man presented for medical evaluation in January 2020 due to pain and a noticeable deformity in the left shoulder. His symptoms initially manifested in 2018, prompting a computed tomography (CT) scan in 2019 at his local healthcare facility. However, the imaging findings did not reveal any pathological abnormalities.

In January 2020, a trephine biopsy of the soft tissue mass was performed, followed by comprehensive histological and immunohistochemical analyses. A diagnosis of high-grade B-cell lymphoma with a proliferative activity index (Ki-67) of 90% was established.

Subsequently, in February 2020, the National Medical Research Center (NMRC) for Hematology conducted a reevaluation of the histological material (see Table 1) and confirmed the diagnosis of high-grade B-cell lymphoma, not otherwise specified (NOS). According to the Hans immunohistochemical algorithm, the lymphoma was classified as the germinal center B-cell (GCB) subtype.

**Figure 1:**
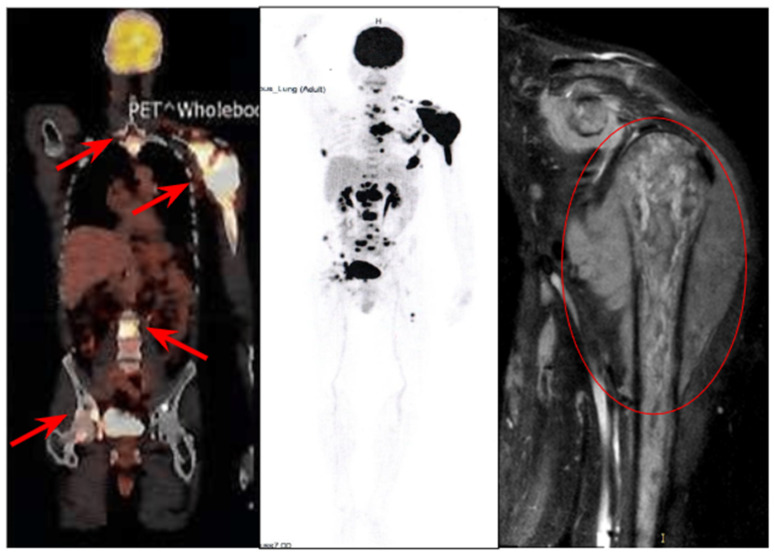
Patient’s baseline 18F-fluorodeoxyglucose (FDG)—positron emission tomography (PET) with computed tomography (CT), February 2020. Computed tomography (CT) provides detailed visualization of the soft tissue mass surrounding the left shoulder, which is outlined with a red line. PET images demonstrate multiple lesions, including involvement of the articular process of the left scapula, a mass in the 5th intercostal space with vertebral bone destruction and extension into the spinal canal, multiple cervical lymph node lesions, and widespread bone metastases.

According to 18F-FDG PET-CT data, the disease exhibited significant localized spread, with a soft tissue mass surrounding the left shoulder measuring 104 × 110 mm (SUVmax 26.38) (Figure 1).

The lesion involved the articular process of the left scapula and distant metastatic sites were also detected, including a mass in the fifth intercostal space with bone destruction of the vertebrae and extension into the spinal canal, multiple cervical lymph node lesions, and bone metastases. Tumor cells—neuroleukemia—were identified in the cerebrospinal fluid. Based on the International Prognostic Index (IPI) assessment, the patient was classified as being at an intermediate risk (IPI = 3).

In accordance with the recommendations of the N.N. Blokhin National Medical Research Center of Oncology under the Russian Ministry of Health, the patient was administered one cycle of R-CHOP at a local medical facility. This treatment led to cerebrospinal fluid sanitation and a reduction in tumor size, vertebral destruction, and the alleviation of pain symptoms. From March to July 2020, the patient underwent five courses of R-EPOCH and seven lumbar punctures involving the administration of methotrexate, cytarabine, and dexamethasone.

After that, the disease progressed, characterized by an enlargement of the mass surrounding the left shoulder joint and the development of severe pain. The patient sought treatment at the NMRC of Hematology, where he was given a pre-phase regimen consisting of cyclophosphamide and dexamethasone. This was followed by a course of R-DHAP combined with methotrexate, lenalidomide, ibrutinib, and venetoclax that was integrated into the therapeutic protocol.

**Figure 2:**
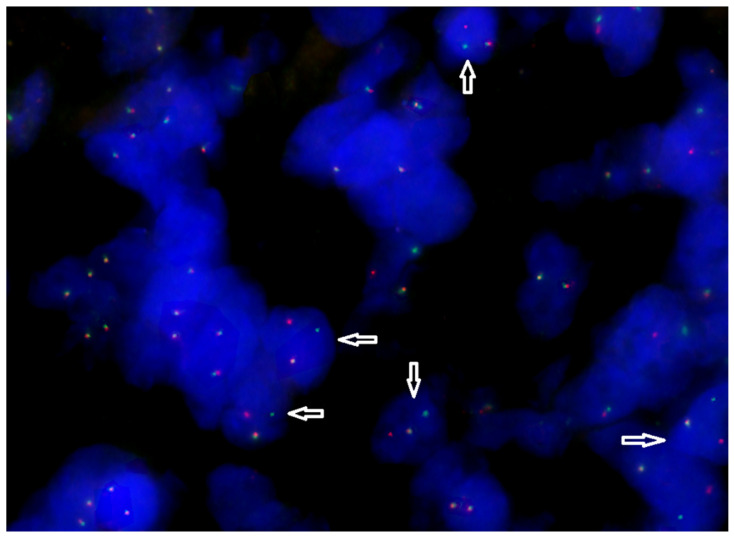
FISH analysis using *BCL2* Break Apart probe (MetaSystems, Germany) (3′ *BCL2* region: red; 5′ *BCL2* region: green). For the analysis, a tissue section from a formalin-fixed, paraffin-embedded (FFPE) tumor biopsy sample was used.

A repeat biopsy of the tumor was performed, revealing the loss of the short arm of chromosome 17 through an FISH analysis (Figure 2). Additionally, PCR followed by Sanger sequencing of exons 7, 8, and 9 of the TP53 gene identified a missense mutation in exon 7 (p.C238G).

Nuclei with *BCL2* rearrangement (indicated by arrows) contain one fused green/orange signal of the *Bcl2* locus on normal chromosome 18 and separated orange (on derivative chromosome 18) and green (on its derivative chromosome partner) signals as a result of translocation.

The therapy initially showed short-term positive effects; however, upon the discontinuation of cytostatic drugs, the disease rapidly progressed. In August 2020, targeted therapy was resumed with lenalidomide, venetoclax, and dexamethasone. Ibrutinib was reintroduced, and nivolumab, a PD-1 immune checkpoint inhibitor, was added to the treatment regimen. Despite this, tumor progression continued, necessitating the initiation of a new chemotherapy regimen incorporating previously unused agents: ifosfamide, dacarbazine, dexamethasone, mitoxantrone, and obinutuzumab.

In October 2020, the patient underwent haploidentical hematopoietic stem cell transplantation (haplo-HSCT) with 5.8 million/kg CD34+ cells transfused. After 23 days, a molecular genetic analysis of peripheral blood confirmed 100% donor chimerism. However, two weeks later, an MRI of the left shoulder joint revealed further tumor growth. To reduce the tumor burden, dexamethasone therapy was administered. Despite these efforts, the patient succumbed to uncontrolled infectious complications and disease progression in October 2020.

A brief timeline of the patient’s treatment is presented in Table 2:

### 2.2. Retrospective Assessment of the Tumor Molecular Profile

The molecular profile of the tumor was analyzed using exome sequencing of clinically significant genes. DNA from a formalin-fixed, paraffin-embedded (FFPE) tumor biopsy sample and paired normal tissue (blood) was used for the analysis. Previously, the tumor sample underwent an assessment by a pathologist and quality control, and the tumor purity was determined to be high (~84%).

A bioinformatics analysis was conducted to identify key genomic alterations in the tumor, including single-nucleotide variants (SNVs), insertions and deletions (Indels), and somatic copy number alterations (SCNAs) [16]. Additionally, microsatellite instability (MSI) [17] and tumor mutational burden (TMB) indices [17,18] were calculated, and germline variants in genes from the ACMG SF v3.2 list were assessed [19]. A bioinformatics analysis was performed in accordance with the recommendations of GDC (Genomic Data Commons) [20]. The alignment to the reference genome, GRCh38.d1.vd1, and read harmonization were performed using bwa [21] and samtools [22], as well as Picard2 v3.3.0 (http://broadinstitute.github.io/picard/ (accessed on 10 October 2024)), IndelRealigner, and BaseRecalibrator from the GATK toolkit v4.6.1.0 [23]. The aligned and co-cleaned BAM files are processed as part of the somatic mutation calling workflow as tumor-normal pairs. Variant calling is performed using separate Mutect2 (GATK v4.6.1.0) [24] and Varscan2 v2.4.2 [25] pipelines. The overall detection threshold for variants is set at 10%, based on the variant allele frequency (VAF) in the sample. The analysis included filtering of identified variants using population databases ExAC and gnomAD [26], with a cutoff threshold of 0,01 to exclude common polymorphisms and focus on potentially pathogenic mutations. Copy number alterations were reported with adjustments for tumor purity and ploidy. The analysis was performed using the tool MSIsensor-pro v1.3.0 (https://github.com/xjtu-omics/msisensor-pro (accessed on 10 October 2024)), with a threshold of 20% for tandem repeat instability. The TMB was calculated as the total number of nonsynonymous mutations per coding region of the tumor genome. Using paired normal tissue, inherited genetic variants were filtered out, and the VCF file generated by Mutect2 was utilized. The analysis was performed using the tool (TMB v1.0) available at https://github.com/bioinfo-pf-curie/TMB (accessed on 10 October 2024) [18].

The clinical results were interpreted using a semi-automated approach, which involves variant prioritization using CancerVar v1.1.2 (https://github.com/WGLab/CancerVar (accessed on 10 October 2024)) [27], followed by a manual review by an expert. This process is supported by publicly available databases such as ClinVar [28], COSMIC [29], OncoKB [30], CIVIC [31], cBioportal [32], PharmGKB [33], and the literature from PubMed [34].

As a result of comprehensive genomic profiling, the following clinically significant somatic mutations were identified (Table 3):

Based on the list of mutations and the BCL2/IGH translocation, the LymphPlex algorithm (https://kylinmu.shinyapps.io/LymphPlexR/ (accessed on 10 October 2024)) classified the tumor’s molecular subtype as “EZB-like without MYC rearrangement”. In DLBCL/HGBL tumors, the number of significant somatic mutations identified is relatively high. Most of the genes listed belong to various molecular genetic subtypes, such as A53, MCD, ST2, and EZB-like [14].

It is important to note that mutations in these genes rarely occur as co-mutations in a single patient (cBioportal database) [32]; rather, they serve as key markers for classification into specific molecular genetic subgroups.

The total tumor mutational burden (TMB) was calculated at 10.67 mutations per megabase (mut/Mb), classifying the tumor as TMB-High (>10 mut/Mb) [35,36,37]. Its microsatellite instability (MSI) status was assessed as MSS (microsatellite stable) [17].

An analysis of copy number variations (CNVs) confirmed the loss of the short arm of chromosome 17 (del(17p)), resulting in loss of heterozygosity of the TP53 mutation. This molecular alteration leads to a complete loss of TP53 protein expression. Additionally, high focal amplification of the MUC3A gene was detected, with an additional 16 copies relative to the diploid genome.

## 3. Discussion

In this case, the initial clinical data, histomolecular subtype, and immunophenotype of the neoplasm indicate an aggressive, treatment-refractory disease with a poor prognosis and unfavorable patient outcome. Among the clinical factors, it is particularly important to note the initial involvement of the central nervous system (CNS). There is limited data on the incidence of CNS involvement in HGBL, NOS; however, recent findings suggest that while rare, CNS involvement is a strong negative prognostic factor, increasing the risk of future CNS relapse and poor survival [38].

It has been established that HGBL, NOS is closer to the most aggressive DLBC/HGBL histotype with rearrangements of MYC, BCL2, and/or BCL6 [39]. According to the WHO definition, HGBL, NOS tumors cannot contain multiple translocations in the MYC, BCL2, or BCL6 genes. Indeed, approximately half of patients with HGBL, NOS carry a single translocation [4,39]. In this case, the patient also had a BCL2/IGH translocation (Table 1).

Initially, as part of standard molecular profiling, a deletion of the short arm of chromosome 17 and a TP53 gene mutation (TP53 p.C238G/del(17p)) were identified. Mutations in TP53 and/or 17p deletion—which affects a cluster of tumor suppressor genes, including TP53—are well-known biomarkers of aggressive disease progression. These alterations are associated with widespread tumor dissemination, including the involvement of the CNS and bone marrow, as well as multidrug resistance to systemic treatment and poor overall and relapse-free survival in B-cell lymphomas, particularly HGBLs [38,40]. 

Comprehensive tumor exome sequencing identified a wide spectrum of significant somatic mutations (SNV/Indels) in B2M, STAT6, STAT3, TREX1, and CREBBP (Table 3), in addition to the previously mentioned alterations.

Mutations in the B2M gene (p.L12R) are one of the most common alterations found in various malignancies, including non-Hodgkin’s lymphomas [32]. This mutation leads to loss of function of β2-microglobulin, a protein essential for stabilizing the trimeric MHC-peptide complex (MHCp) on the cell surface [41,42]. As a result, the presentation of tumor neoantigens via MHC class I is disrupted, allowing the tumor to evade immune surveillance [41]. 

Gain-of-function mutations in STAT6 and STAT3 are frequently observed in B-cell lymphomas. The JAK-STAT pathway plays a crucial role in treatment resistance in both hematologic and solid malignancies. The oncogenic hotspot mutation STAT6 p.D419G, found in this case, affects the DNA-binding domain of the protein and is commonly associated with relapsed or refractory GCB subtype DLBCL [43].

Moreover, mutations in STAT3 and STAT6 are known to increase PD-L1 expression and enhance the production of tumor-associated antigens [44]. Specifically, the STAT3 p.S614R mutation has been linked to high PD-L1 expression in lymphomas [45]. However, persistent STAT3/STAT6 activation also contributes to T-cell exhaustion and dysfunction within the tumor microenvironment, posing a significant challenge for immunotherapy [43,46,47]. Combining JAK-STAT inhibitors with anti-PD-1 therapies has been proposed as a potential strategy to overcome this resistance.

In the presented clinical case, a somatic nonsense mutation in the TREX1 gene (p.T49fs) was identified for the first time. In the literature, it is described exclusively as germline, associated with Aicardi–Goutieres syndrome, which is a severe form of hereditary encephalopathy and interferonopathy (AGS1) [48,49]. There are no data on clinical cases of DLBCL/HGBL inactivating the TREX1 mutation. TREX1 dysfunction affects intracellular metabolism and the efficient utilization of cytosolic DNA, causing nucleic acid accumulation in the cell, which provokes an autoimmune response and high IFN1 production [50]. In particular, this mechanism is important for efficient DNA degradation in dying tumor cells that have undergone chemotherapy [51,52]. The loss of TREX1 results in the overactivation of cGAS-STING, which triggers the type I interferon response [53,54,55]. Researchers are trying to use this mechanism to activate IFN1-dependent antitumor immunity [54]. STING antagonists for tumors with inactivated TREX1 are considered as potential effective therapeutic agents, and patients with somatic mutations in this gene may be candidates for inclusion in clinical trials of these drugs [56].

Loss-of-function mutations in the epigenetic modifier CREBBP are common oncogenic drivers in B-cell lymphomas, particularly in the GCB subtype [57,58]. In this clinical case, a previously undescribed somatic nonsense mutation of CREBBP, p.C367Ter, was identified. The CREBBP protein consists of 2442 amino acids, and a stop-gain mutation disrupts its chain at the onset within the region of the TAZ-type 1 domain (zinc finger) (https://www.uniprot.org/uniprotkb/Q92793/entry (accessed on 14 January 2025)). CREBBP mutations result in a defect in the chromatin modifier protein, which is involved in many cellular processes and functions as a transcriptional cofactor and histone acetyltransferase. In solid tumors, their significant correlation with TMB-High and/or MSI-High status and high PD-L1 expression has been noted [59,60].

Among the significant copy number changes, a high focal amplification (+16 copies) of the MUC3A gene encoding highly glycosylated membrane-bound mucin was detected. Similar amplifications have been associated with increased metastasis and recurrence in colorectal cancer and a poor prognosis [61].

In the described clinical case, the list of significant somatic events is quite broad and includes mutations that are characteristic for different molecular genetic subtypes of DLBCL/HGBL (Table 3) [14]. However, it is a generally accepted principle that within a single case of DLBCL/HGBL, oncogenic hits that involve the same pathways are generally mutually exclusive [62]. Using the simplified LymphPlex algorithm (https://kylinmu.shinyapps.io/LymphPlexR/ (accessed on 10 October 2024)), the described tumor was classified as “EZB-like without MYC rearrangement group” rather than “TP53mut”. This was due to the presence of a rearrangement of the BCL2 gene. It can be assumed that the assignment of this tumor to a specific molecular genetic subgroup in this case may be difficult due to the significant overlap of molecular markers from different subtypes.

A commonly used indicator of tumor genomic instability is its tumor mutational burden (TMB), which is widely implemented in the molecular analysis of various solid tumors [36]. The TMB is defined as the number of non-synonymous mutations per megabase (Mb) in the coding regions of the tumor genome. High levels of this indicator are associated with an improved response to treatment with immune drugs and overall relapse-free survival in the context of the use of immune checkpoint inhibitors in many types of cancer. The standard threshold value used in clinical practice is usually set at 10 mut/Mb [35,63], but it may vary in studies. For example, results from a prospective study indicate that the median TMB among patients with DLBCL was 3.6 Mut/Mb, and the threshold value for determining the TMB-High status was calculated and set at 4.5 mut/Mb. A high mutational burden was significantly associated with better event-free and overall survival rates for DLBCL cases with IPI 3-5 [37].

In this clinical case, when calculating the TMB, a value of 10.67 mut/Mb was obtained, which corresponds to the status of “TMB-high”. The disease had an aggressive course; the tumor was refractory to both standard regimens and various attempts to use “off-label” therapy. The patient was treated with the anti-PD-1 immunotherapy drug (nivolumab) against the background of targeted therapy with lenalidomide, venetoclax, and ibrutinib, but the disease progressed and the tumor continued to grow. Despite the high mutational burden, in this case, a significant number of biomarkers indicate the potential ineffectiveness of immunotherapy with immune checkpoint inhibitors, such as B2M, TP53/del(17p), STAT3, and STAT6. TP53 is a well-known biomarker of a poor prognosis and unfavorable response to ICI, B2M loss leads to impaired antigen presentation via MHC, and STAT3 and STAT6 are responsible for creating an immunologically exhausted tumor microenvironment [41,43,46,47,64,65]. The identification of an inactivating somatic mutation in TREX1 in DLBCL/HGBL may prompt a retrospective analysis of existing omics data from DLBCL/HGBL patient samples, potentially leading to the organization of clinical trials evaluating STING agonists in the context of these diseases [66,67,68]. Against the background of the formed violation of the presentation of MHC neoantigens, the depletion of the tumor microenvironment, and the activation of innate immunity mechanisms, the use of STING agonists may be another solution in the future when choosing immunotherapy. Further studies are needed to establish the predictive and prognostic significance of the described biomarkers for these malignancies.

## 4. Conclusions

Thus, the comprehensive genomic profiling of this HGBL, NOS case revealed significant somatic alterations beyond standard biomarkers, along with a high tumor mutational burden. These findings help explain the ineffectiveness of both standard systemic treatments and PD-1 inhibitor-based immunotherapy. Given the ongoing research into STING agonists and the role of TREX1 inactivation in cGAS-STING signaling across various malignancies, the TREX1 mutation identified here may serve as a valuable target for developing new immunotherapy strategies.

## Figures and Tables

**Table 1:** HGBL immunophenotype and results of IHC and FISH karyotyping.

Marker	Meaning
CD20+	Intense membrane expression
CD10+	Intense cytoplasmic reaction
BCL6+	Intense nuclear reaction
BCL2+	Intense cytoplasmic reaction
Ki-67	90%
MUM1+	Single small cells
CD23	Reaction with CD23 antibody is questionable (negative/false negative?)
CD3+, CD5+, CD43+	Few small lymphoid T-cells
c-MYC+	70–80% positive lymphoid infiltration cells (heterogeneous nuclear reaction)
TdT-	no TdT positive cells detected
GCB/non-GCB subtype	GCB subtype
Double-expressor (DEL) phenotype	Yes
Translocations (FISH)	*BCL2* rearrangement and 17p13/*TP53* deletion were detected; *MYC*, *BCL6* rearrangements were not detected.
Karyotype (bone marrow)	46, XY. Clonal chromosomal aberrations were not detected.

**Table 2:** Patient’s treatment timeline.

Date	Treatment	Result
03.2020–07.2020	R-CHOP №1, R-EPOCH №5	Progression
08.2020	R-DHAP, methotrexate, lenalidomide, ibrutinib, venetoclax, nivolumab	Progression
09.2020	ifosfamide, dacarbazine, dexamethasone, mitoxantrone, obinutuzumab	Progression
10.2020	haploidentical hematopoietic stem cell transplantation (haplo-HSCT)	Progression. Death

**Table 3:** Pathogenic somatic mutations identified by comprehensive genomic profiling (SNV/Indels).

Gene	Mutation	Classification	OncoKB [30]	COSMIC [29]	ClinVar [28]	AF (%)/Read Depths	Function	LOH
TP53	NM_000546.6(TP53):c.712T>G (p.Cys238Gly)	Pathogenic	Likely Oncogenic Likely LOF Level 1	COSV52711932	Yes	75%/16x	LOF	LOH
B2M	NM_004048:exon1:c.T35G:p.L12R	Probably pathogenic	Likely Oncogenic Unknown Biological Effect	COSV62563197	-	92,3%/46x	LOF	-
STAT6	NM_003153:exon12:c.A1256G:p.D419G	Probably pathogenic		COSV55668829	Yes	66,7%/27x	GOF	-
STAT3	NM_213662:exon20:c.C1842G:p.S614R	Pathogenic	Likely Oncogenic Likely GOF Level 3	COSV52888203	-	25%/52x	GOF	-
TREX1	NM_033629.6(TREX1):c.144dup (p.Thr49fs)	Pathogenic	-	-	Yes	40%/62x	LOF	-
CREBBP	NM_004380:exon4:c.T1101A:p.C367X Stopgain	Probably pathogenic	-	-	-	50%/24x	LOF	-

Note: LOF—Loss-of-function; GOF—Gain-of-function; LOH—Loss of heterozygosity.

## Data Availability

The raw data supporting the conclusions of this article are available from the authors upon request, subject to privacy and ethical restrictions.
